# Formation and Stability of Bulk Carbonic Acid (H_2_CO_3_) by Protonation of Tropospheric Calcite

**DOI:** 10.1002/cphc.201200422

**Published:** 2012-06-15

**Authors:** Juergen Bernard, Markus Seidl, Erwin Mayer, Thomas Loerting

**Affiliations:** a Institute of Physical Chemistry, University of InnsbruckInnrain 52a, 6020 Innsbruck (Austria); b Institute of General, Inorganic and Theoretical ChemistryUniversity of Innsbruck (Austria)

**Keywords:** atmospheric chemistry, carbonic acid, gas-phase chemistry, IR spectroscopy, mineral dust

Organic acids play an important role in the acidification of our atmosphere. These weak acids can contribute up to 60 % of the free airborne acidity. By far the most abundant organic acids are the C1 and C2 monocarboxylic acids, formic acid (HCOOH) and acetic acid (CH_3_COOH), which show mixing ratios in the gas phase ranging up to 20 ppb over land^1^,^2^ and down to 0.2 ppb in the remote oceanic boundary layer or troposphere.^3^,^4^ These acids are partitioned between the gas phase and the particulate phase, where roughly one half to two thirds can be found in particulate matter (PM2.5).^2^ The most important removal mechanism is dry deposition, which accounts for more than 90 % of the total organic acid deposition budget. The remaining fraction is removed by rain as particulate-phase acids, whereas removal by chemical reactions is negligible.^1^,^5^ In addition to the two most important organic acids, C3–C10 aliphatic monocarboxylic acids^1^ and C2–C11 aliphatic dicarboxylic acids^1^,^2^,^6^–^11^ as well as aromatic carboxylic acids^1^ have also been observed in air. The water-soluble fraction of organic carbon can on average consist of 35 % mono- and dicarboxylic acids.^12^ While the C2-dicarboxylic acid, oxalic acid (COOH)_2_, is commonly observed in all field studies, the C1-dicarboxylic acid, carbonic acid (H_2_CO_3_), has barely received any attention, mainly because it is thought that it immediately decomposes to water and carbon dioxide. However, it has previously been shown that gaseous, water-free carbonic acid is surprisingly stable,^13^ that amorphous and crystalline solids of pure carbonic acid can be produced and stored without decomposition at temperatures up to 230 K even in the presence of water^14^ and that this solid can be sublimed at, for example, 220 K and recondensed in vacuo at surfaces of lower temperature.^15^

Formation of bulk carbonic acid in the laboratory has so far been achieved 1) by high-energy irradiation of CO_2_ or CO_2_/H_2_O mixtures,^16^–^20^ 2) through surface reactions of CO molecules with hydroxyl (OH) radicals at 10–40 K^21^ and 3) by protonation of aqueous or methanolic solutions of KHCO_3_ or K_2_CO_3_ at low temperatures of ∼140–180 K.^22^–^25^ Mechanism (1) is believed to be of astrophysical relevance, and carbonic acid is hence supposed to be present, for example, on comets, the Galilean satellites, on Venus and on the Martian surface.^15^,^18^,^26^–^33^ For mechanisms (2) and (3) no relevance in nature, and in particular in the Earth’s atmosphere, is envisioned because in the case of (2) temperatures are too low and in case of (3) aqueous/methanolic solutions of potassium(bi)carbonate are not present in the atmosphere at *T*<180 K. A possible mechanism (4) of carbonic acid formation in nature was outlined by Grassian et al., who studied the interaction of gas-phase acids such as formic acid, acetic acid, sulphur dioxide and nitric acid with calcium carbonate particles (CaCO_3_) at ambient temperature. Under dry conditions of <1 % relative humidity these acids react with the Ca(OH)(CO_3_H) surface layer of calcium carbonate and produce *surface-adsorbed* carbonic acid, which is stable even at 296 K under dry vacuum conditions, but decomposes at higher relative humidities.^34^–^36^ According to this mechanism, carbonic acid is an important, but short-lived intermediate in the surface chemistry of calcium carbonate even at room temperature.

Here, we propose a novel mechanism (5), which produces *bulk* carbonic acid under conditions relevant to our atmosphere. This mechanism involves protonation of mineral dust particles, such as CaCO_3_, in the troposphere by acids, for example droplets containing HCl, at ≥200 K. Using FT-IR spectroscopy we demonstrate that high-surface-area CaCO_3_ particles are consumed on a time scale of hours at 200 K in a humid atmosphere, while a non-volatile component, possibly Ca(HCO_3_)_2_, and aqueous/amorphous H_2_CO_3_ (that crystallizes at 220 K to the β-polymorph of carbonic acid) are produced. By contrast, as shown by Santschi et al.,^37^ reaction of CaCO_3_ with HCl at 300 K involves Ca(OH)(HCO_3_) and presumably CaCl_2_⋅2H_2_O, but not carbonic acid and not Ca(HCO_3_)_2_. Furthermore, we show that H_2_CO_3_ does not decompose readily even at 250 K in a humid atmosphere (0.6–0.8 mbar water vapour pressure, ≍60–100 % relative humidity, ≍100–450 mbar total pressure). At higher temperatures H_2_CO_3_ sublimes and/or decomposes. We therefore suggest that some of the CaCO_3_ and also the MgCO_3_ fraction of mineral dust, such as Saharan or Asian dust, is converted in the mid- and high troposphere at 200–250 K to aqueous/amorphous H_2_CO_3_ and may even crystallize and exist as H_2_CO_3_, more precisely as the β-polymorph.^22^–^25^ Because of the long-term stability of H_2_CO_3_ at conditions relevant to the troposphere we suggest that carbonic acid contributes to the acidity in the troposphere.

Figure 1 depicts the IR spectrum of the CaCO_3_ powder in the optical window of the range of 4000–500 cm^−1^. The spectrum clearly shows calcite bands, in particular *ν*_4_(E′) at 713 cm^−1^, *ν*_2_(A_2_′′) at 877 cm^−1^ and the broad and strong *ν*_3_(E′) centred at 1422 cm^−1^. This agrees well with literature data.^38^,^39^ In addition, smaller absorptions, including overtones and combination bands, can be seen, for example at 1795 cm^−1^, 2511 cm^−1^ and 2874 cm^−1^. The sloping baseline between 4000 and 2000 cm^−1^ is characteristic of wavelength-dependent scattering of small particles.^38^ No ethanol bands can be discerned in Figure 1, which indicates that the calcite sample is dry after the procedure of transferring the powder onto the ZnSe window.

**Figure 1:**
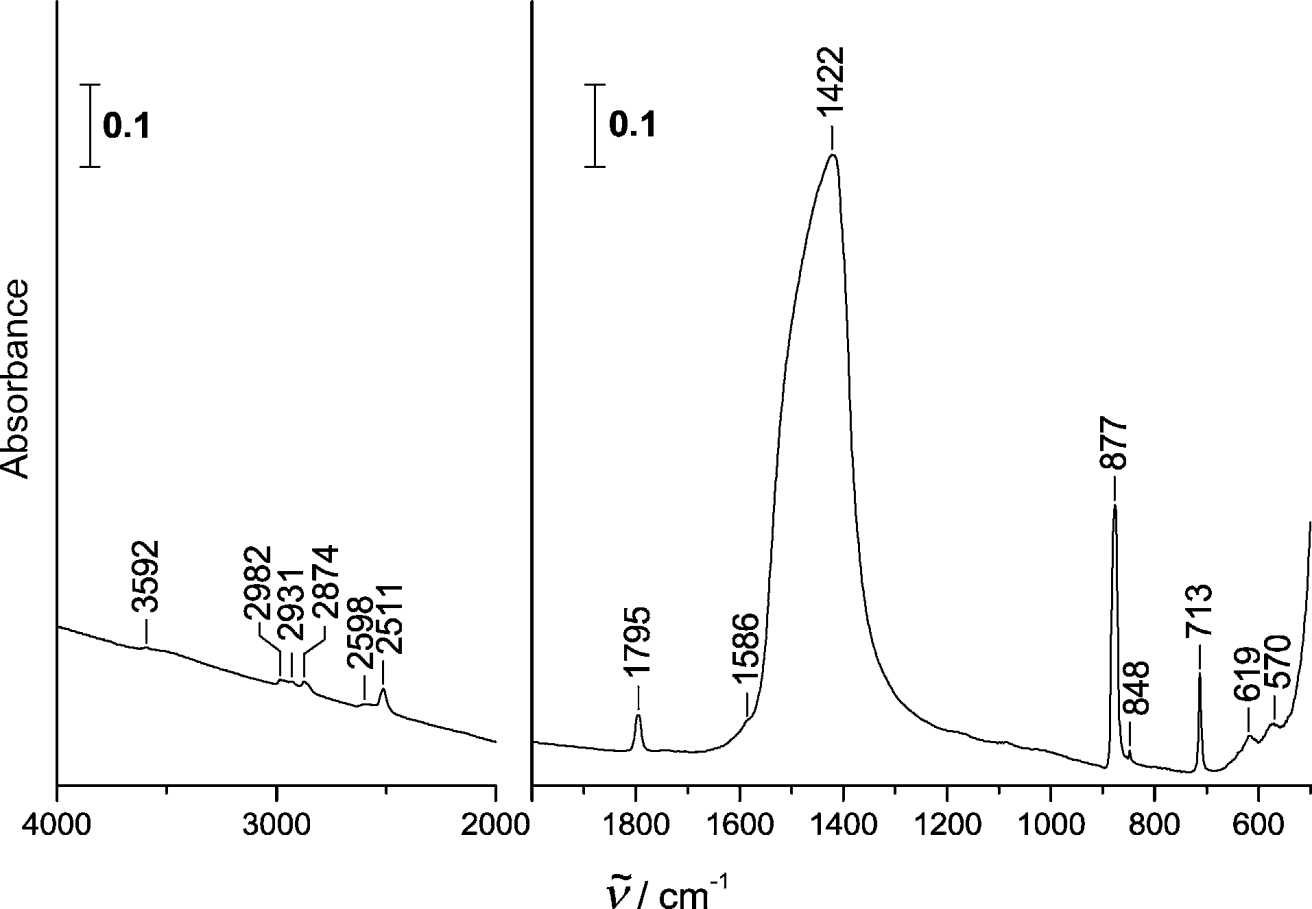
FT-IR spectrum of ≍1 mg high-surface-area calcite (CaCO_3_) particles of equivalent spherical diameter of 600 nm^38^ on a 25×4 mm ZnSe optical disc.

Figure 2 a shows FT-IR spectra after treatment with aqueous HBr at 82 K, introduction of a 10.7 mbar inert atmosphere, heating to 200 K and remaining at 200 K for 60 min. Spectrum (1) was recorded immediately before pumping off the atmosphere, spectrum (2) immediately after pumping off the atmosphere. While calcite absorptions can still be seen in this spectrum (e.g. 1791/1417/875), new bands clearly appear at 1722 cm^−1^, 1630 cm^−1^, 1471 cm^−1^, ∼1300 cm^−1^ and 801 cm^−1^. The new bands agree well with the band positions reported for amorphous carbonic acid (1721/1476/1306/803) in ref. 14 [Figure 1, spectrum (1) therein]. All strong absorptions reported by Winkel et al.^14^ also appear here and so we conclude that amorphous carbonic acid was formed. In addition, we attribute the band at 1630 cm^−1^ to the hexahydrate of HBr.^40^–^43^ Spectra (3) and (4) show that calcite disappears within 71 min at 200 K, that is, the absorptions at 1791/1417/875 disappear slowly and simultaneously. After 71 min all the calcite bands are very weak, indicating that most of the calcite has disappeared. In addition some peaks also sharpen. In particular, a sharp band at 1634 cm^−1^ develops from the broader one at 1630 cm^−1^. We attribute this to crystallization of the HBr hexahydrate from the amorphous phase. Water is pumped off only to a small extent at 200 K within 71 min, as indicated by the broad OH stretching band centered at ∼3300 cm^−1^, which remains largely unaffected.

**Figure 2:**
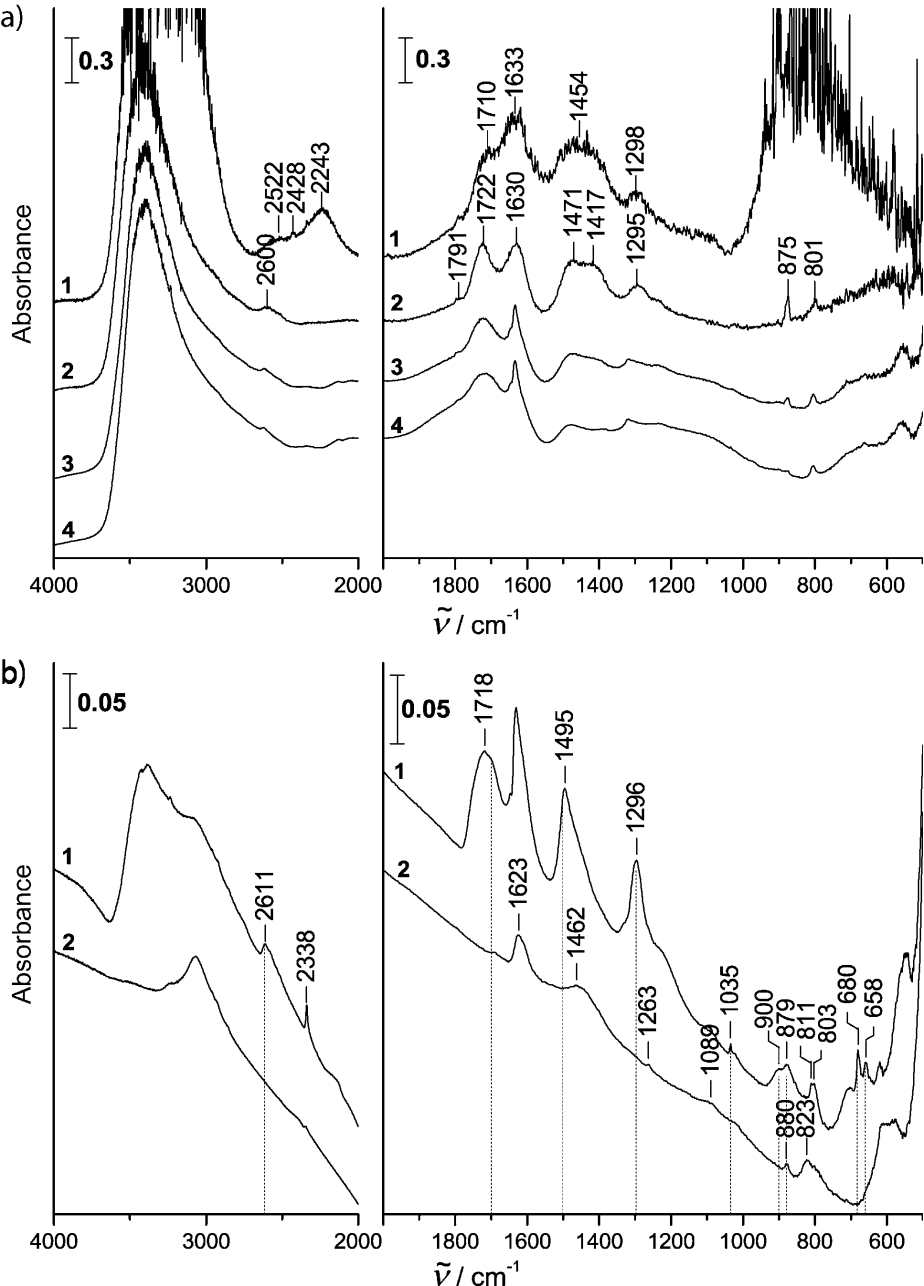
a) FT-IR spectra indicating formation of reaction products (amorphous H_2_CO_3_ and HBr⋅6H_2_O) after spraying a layer of 4 m HBr at 82 K onto the calcite sample in a vacuum chamber and heating the sample to 200 K and annealing for 60 min at 200 K in a helium atmosphere of 11 mbar (1), after pumping off the helium atmosphere to 1×10^−3^ mbar (2), after 16 min at 200 K (3) and after 71 min at 200 K (4). b) FT-IR spectra indicating crystalline β-H_2_CO_3_ in the product mixture after 70 min at 220 K and 12 min at 230 K (1) and non-volatile component remaining after removal of β-H_2_CO_3_ by keeping the sample overnight in vacuo at 300 K (2).

This broad band disappears, however, after 70 min at 220 K and 12 min at 230 K [see spectrum (1) of Figure 2 b], which mimics the evaporation of water at low humidity in the troposphere. At the same time the amorphous carbonic acid crystallizes to the β-polymorph. Winkel et al. have established the following criteria indicating the crystallization of β-H_2_CO_3_ (see Figure 1 in ref. 14): 1) growth of a doublet at 661 cm^−1^ and 683 cm^−1^ at the cost of a broad band at 649 cm^−1^; 2) appearance of a broad band with a double-maximum at 880 cm^−1^/900 cm^−1^; 3) growth of a sharp band at 1035 cm^−1^ from a broad band at 1020 cm^−1^; 4) appearance of a single band at 1297 cm^−1^ from a structured band with a maximum at 1267 cm^−1^; 5) shift and sharpening of a broad band from 1476 cm^−1^ to 1501 cm^−1^; 6) changes in a structured band at 1721 cm^−1^ to a differently structured band at 1700 cm^−1^ and 7) shift and sharpening of a broad band from 2555 cm^−1^ to 2616 cm^−1^. All these criteria, except maybe for the shift in ν(C=O), are clearly evident in spectrum (1) in Figure 2 b and are marked by vertical lines. That is, we infer crystallization to β-H_2_CO_3_ at 220 K. In addition, the crystalline hexahydrate of HBr is still present in the sample. Some additional features of the spectrum can be discerned. The sharp band at 2338 cm^−1^ is attributed to trapped CO_2_. The asymmetric shape of the HBr hexahydrate band at 1634 cm^−1^ and of the carbonic acid bands at 1495 and 1296 cm^−1^ indicate the presence of a trace of an additional, unidentified species. This species can be separated from carbonic acid and the HBr hexahydrate, because, in contrast to them, it is a non-volatile species. After heating to 300 K and leaving the sample in vacuo overnight, CO_2_, carbonic acid and the HBr hexahydrate are pumped off, and the non-volatile species remains. The spectrum of this species with bands at 1623/1462/1263/1089/880/823 is shown as trace 2 in Figure 2 b. The *ν*(C=O) at 1623 and the π(CO_3_) at 832 is close to the *ν*(C=O) and π(CO_3_) observed in KHCO_3_ (1618/832).^44^ However, other bands are shifted: 1462 vs 1405, 1089 vs 1001, 610 vs 690. We speculate that the species remaining in the window is Ca(HCO_3_)_2_, which crystallizes as an alkaline-earth bicarbonate in a space group different from the one known for alkali bicarbonates such as KHCO_3_. That is, calcite dust is converted by freeze-concentrated aqueous acid droplets to amorphous carbonic acid after an hour at 200 K, and crystallization of carbonic acid may occur at slightly higher temperatures in low-humidity conditions. Incomplete conversion results in the formation of what we regard to be Ca(HCO_3_)_2_.

Furthermore, we tested the stability of β-H_2_CO_3_ at tropospheric conditions. We produced some β-H_2_CO_3_ by reaction of concentrated HCl (12 m) with an excess of CaCO_3_ at 200 K at ∼100 mbar pressure. We identify excess CaCO_3_, CaCl_2_⋅2 H_2_O and β-H_2_CO_3_ as the products of this reaction. CaCl_2_⋅2 H_2_O bands are observed at ∼3500/1629/1614. Figure 3 a shows the spectra collected after having reached 260 K (1), after 17 min at 260 K (2) and after an hour at 260 K (3). Bands assigned to β-H_2_CO_3_, CaCO_3_ and CaCl_2_⋅2 H_2_O are marked by arrows, stars and open circles, respectively. While the spectra are unchanged after a few hours at 200–250 K, the decomposition of β-H_2_CO_3_ is evident on a time scale of one hour at 260 K and 50–70 % relative humidity. After having been kept for prolonged periods at lower temperatures at 50–70 % relative humidity, it is clear that carbonic acid is still present initially at 260 K, from the presence of the bands marked by the arrows in Figure 3 a, spectrum (1). The difference spectrum between spectra (3) and (1) from Figure 3 a is shown as spectrum (1) in Figure 3 b. This difference spectrum is compared to spectrum (2) in Figure 3 b of crystalline β-carbonic acid (identical to spectrum 4 in Figure 1 of ref. 14). The agreement between these two spectra is excellent, which leads us to conclude that β-H_2_CO_3_ slowly disappears at 260 K and 50–70 % relative humidity, whereas the other components in the mixture (CaCl_2_⋅2 H_2_O and CaCO_3_) remain unaffected. After an hour at 260 K only a minor fraction of the carbonic acid band intensities remains [see spectrum (3) of Figure 3 a), so that the half-life can be estimated to be about 30 min at these conditions. For comparison, two of us calculated the half-life of carbonic acid in the presence of two water molecules to be about 40 minutes at 260 K and about 20 years at 200 K (Figure 2 in ref. 13a). The agreement between these calculations and the experimental findings reported herein suggests that the decomposition mechanism involving carbonic acid and water at a 1:2 ratio is suitable for describing decomposition kinetics at 60% relative humidity. Independent experiments on the stability of *pure* crystalline β-carbonic acid have confirmed this result of slow decomposition at 260 K and 50–70 % relative humidity, but of no significant decomposition at lower temperature. That is, at 240 K and high relative humidity, carbonic acid, once formed from mineral dust, may persist over many hours. At lower humidities and/or temperatures we even expect stability for many days without any significant decomposition.

**Figure 3:**
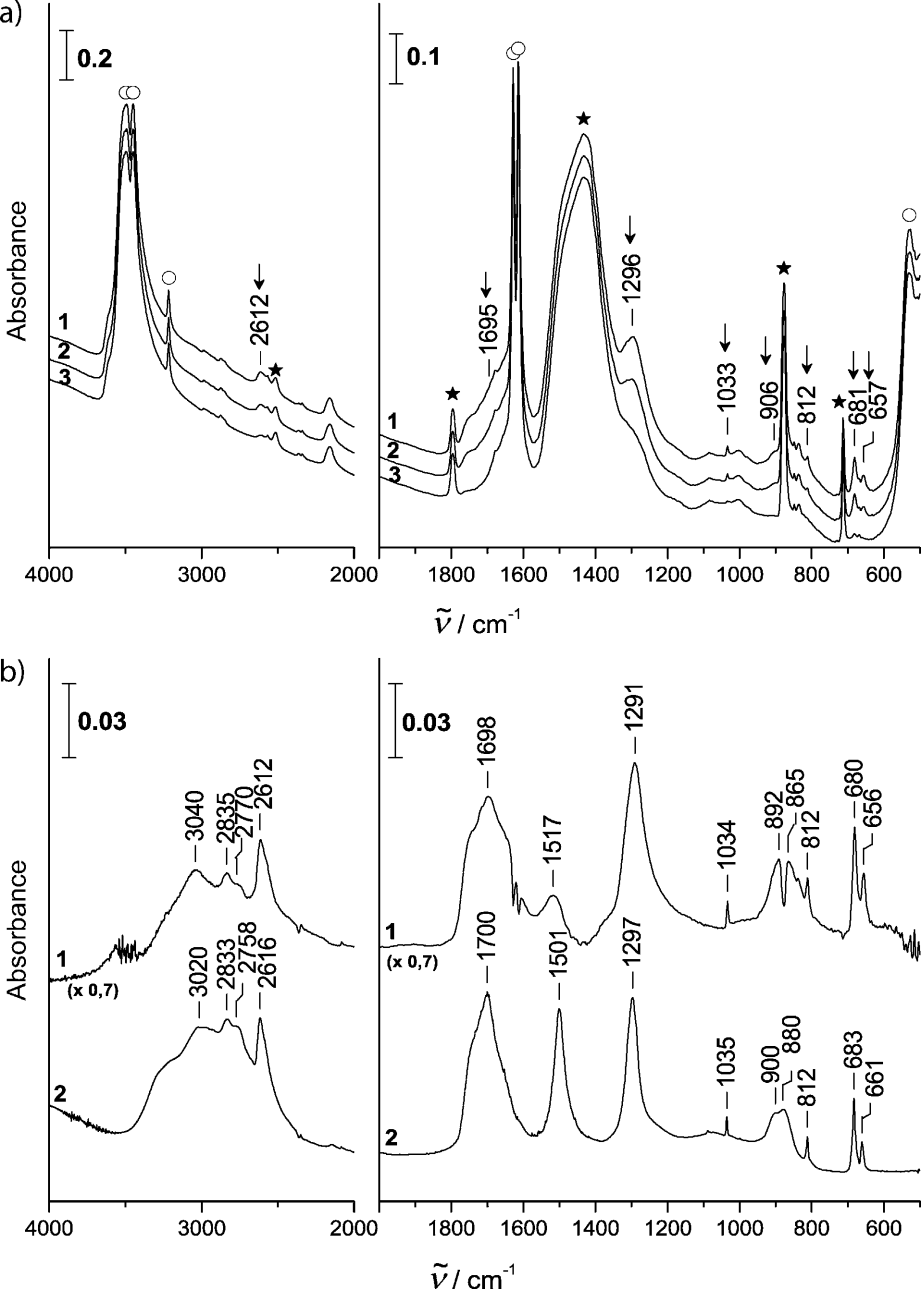
Thermal stability of β-H_2_CO_3_ in the product mixture at 260 K, ∼400 mbar helium and ∼60 % relative humidity. The product mixture was obtained by protonation of high-surface area calcite with ∼12 m HCl at 200 K for 60 min in ∼100 mbar helium at relative humidities exceeding 100 %, i.e., in the presence of condensed ice. The sample was kept for ∼5 h at 200–250 K before heating to 260 K. a) Spectra recorded at 260 K, ∼400 mbar helium and ∼60 % relative humidity. 0 min at 260 K (1), 17 min at 260 K (2) and one hour at 260 K (3). Arrows: β-H_2_CO_3_, stars: CaCO_3,_ circles: CaCl_2_⋅2H_2_O b) Comparison of the spectrum of the disappearing species [calculated as difference spectrum of spectra (3) and (1) of (a)] with the spectrum of crystalline β-carbonic acid (corresponds to spectrum (4) in Figure 1 from ref. 14).

Figure 4 summarizes the experiments reported herein schematically in the context of atmospheric chemistry. The encounter of a mineral dust particle of ∼600 nm diameter, namely CaCO_3_, with concentrated aqueous HCl droplets constitutes step (1). At temperatures and pressures relevant in the troposphere the reaction progresses within about an hour. Some CaCO_3_ remains, while CaCl_2_⋅2 H_2_O, possibly some Ca(HCO_3_)_2_, and amorphous carbonic acid form. At somewhat higher temperatures (∼220–240 K) amorphous carbonic acid crystallizes to β-H_2_CO_3_. We emphasize that treatment of particulate calcite with acids as shown here above 200 K results in the same polymorph obtained previously by high-energy irradiation of CO_2_ or CO_2_/H_2_O ice mixtures^16^–^20^ and by reaction of water-soluble (bi)carbonates with acids in aqueous solution above 160 K,^14^,^15^,^23^,^25^,^27^ whereas other methods involving solvents other than water^22^,^24^ or no solvents^21^ produce different polymorphs. β-H_2_CO_3_ remains stable over prolonged periods below 240 K even at relative humidities of 50–70 %. We notice slow disappearance of β-H_2_CO_3_ only at 260 K, where the half-life is of the order of 30 min at 50–70 % relative humidity, presumably due to decomposition to CO_2_ and H_2_O. In total, CaCO_3_ is consumed by HCl, producing CO_2_, H_2_O and CaCl_2_⋅2 H_2_O. However, at tropospheric temperatures of 200–240 K bulk (amorphous and/or crystalline) solid carbonic acid may exist over prolonged periods of time. We therefore suggest that amorphous and crystalline β-H_2_CO_3_ exists as bulk species in the troposphere containing mineral dust. A proof of this hypothesis would be equivalent to the discovery of this elusive species outside scientific laboratories as bulk species in the environment of the Earth. In order to possibly detect carbonic acid in air samples by field studies, we suggest using contactless techniques, for example, infrared spectroscopy, or alternatively sampling techniques not involving bringing the collected sample to ambient temperature and not involving dissolution of the sample in water in order to avoid decomposition of carbonic acid. By contrast to other organic acids in the atmosphere, carbonic acid decomposes at ambient temperature and in aqueous solution. However, it is surprisingly stable over prolonged periods even in the presence of water vapour at tropospherically relevant temperatures.

**Figure 4:**
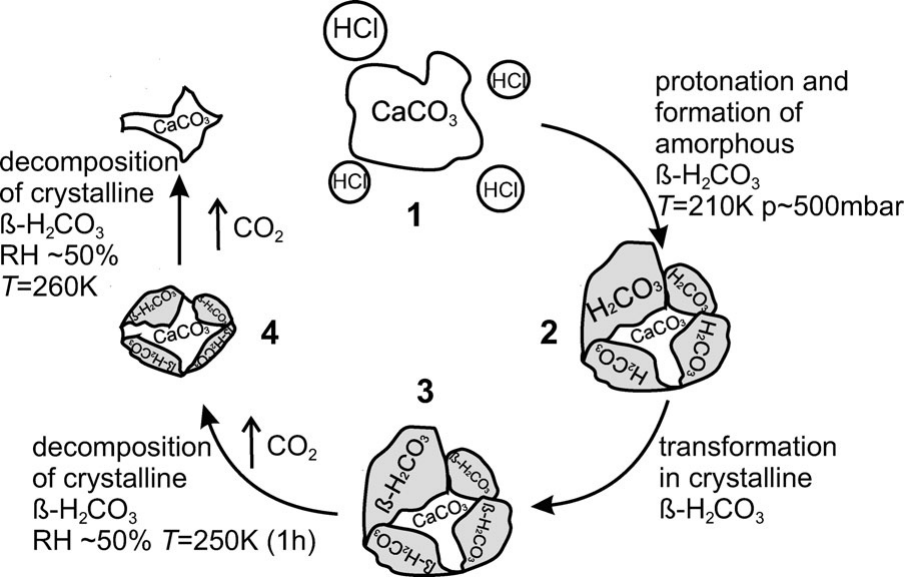
Carbonic acid formation in the atmosphere. Processing of mineral dust particles (CaCO_3_) by highly concentrated aqueous HCl droplets (1). Amorphous carbonic acid forms at *T*=200 K and *p*∼450 mbar (2), and crystallizes at higher temperatures to β-H_2_CO_3_ (3). Decomposition of β-H_2_CO_3_ does not take place at 220 K, a few hundred millibars total pressure and>50 % relative humidity, but takes place with a half-life of 30 min at 260 K and 50–70 % (4).

## Experimental Section

Commercial CaCO_3_ powder (OMYACarb, UF) provided by the group of Vicki Grassian with a manufacturer specified equivalent spherical diameter of 600 nm was used in this study. The BET surface area of this powder was found to be 10.1±0.3 m^2^ g^−1^ using N_2_ gas.^38^ Approximately 1 mg of this powder was immersed in two droplets of ethanol and distributed evenly on a 25 mm diameter optical disc (ZnSe or CsI). After evaporation of ethanol the powder sticks to the optical disc and is mounted in a cryo-sample holder inside a vacuum chamber. The vacuum chamber, pumped to a base pressure of 5×10^−7^ mbar using an oil-free scroll pump (Varian Triscroll 300) and a turbomolecular pump (Leybold Turbovac 361) is equipped with a hygro-thermometer (Qhygo-Temp80) and two 49 mm diameter optical discs (KBr) so that a beam of light can pass through the vacuum chamber and the sample. FT-IR measurements are done using a VARIAN Excalibur 3100 spectrometer at 4 cm^−1^ resolution. Figure 1 shows the FT-IR spectrum of the calcite powder on the optical disc. Two sets of experiments related to possible protonation of calcite are reported herein. The first set (results depicted in Figure 2) deals with the formation of carbonic acid from calcite at 200–220 K. The second set deals with the question of the stability of carbonic acid in a humid environment up to 260 K (Figure 3).

After cooling the cryo-sample holder to 82 K, an airbrush (Harder& Steenbeck Infinity) is used to spray micron-sized droplets of a concentrated aqueous solution of HX (4 m HBr or 12 m HCl) through an orifice (500 μm diameter) into the pumped vacuum chamber, which produces a directed, supersonic beam of droplets onto the sample. This process mimics the encounter of micron-sized acid droplets and calcium carbonate particles in mineral dust in the troposphere. A temperature of 82 K is used for droplet deposition because diffusion and chemical reaction are inhibited, and so droplets and solid particles coexist without reaction^22^–^24^. In order to stress the relevance of this experiment in the context of the chemical processing of mineral dust in the upper troposphere we then disconnected the pumps from the vacuum chamber and introduced an inert atmosphere of 10.7±0.5 mbar into the chamber. The sample temperature was next kept in this atmosphere for an hour at 200 K. The acid droplets are in a semi-frozen, freeze-concentrated state under such conditions, similar to the situation in the troposphere. At this temperature there is significant diffusion, dissolution of calcium carbonate in the droplets and chemical reaction, which is monitored by FT-IR spectroscopy. After processing the calcite particles with acid droplets for one hour the atmosphere was pumped off in order to improve the signal-to-noise ratio of spectra. Please note that direct deposition of the droplets in vacuo at 200 K produces similar spectra, whereas a directed beam of droplets to the sample is not possible in the presence of an atmosphere. In previous publications we reported the formation of β-carbonic acid of water-soluble carbonates (K_2_CO_3_, Cs_2_CO_3_) and bicarbonates (KHCO_3_) with aqueous acids (HCl, HBr)^14^,^15^,^23^,^25^,^27^ at temperatures of 160–240 K in vacuo. That is, we reported reaction between (freeze-concentrated) aqueous solutions of carbonate with (freeze-concentrated) acidic solutions. Here, we study for the first time whether or not reaction also takes place between solid particulate matter, namely a carbonate insoluble in water (CaCO_3_), and (freeze-concentrated) acidic droplets at 200–220 K (Figure 2). In order to obtain quantitative conversion to carbonic acid, a molar ratio HX:CaCO_3_ of 2:1 is necessary according to Equation (1):

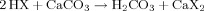


A lower ratio results in incomplete protonation and appearance of the intermediate calcium bicarbonate, which is not known to exist as a solid, but merely in solution. As an example the situation involving a ratio of 1:1 is shown in Equation (2):

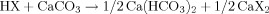


The second set of experiments is aiming at the question whether or not carbonic acid (H_2_CO_3_), once formed by processing of mineral dust in the atmosphere, remains stable without decomposition for a sufficiently long time in the humid troposphere. To this end we studied the reaction of concentrated HCl (12 m) with an excess of CaCO_3_ at 200 K, and the stability of the resulting β-H_2_CO_3_ in the mixture of products up to 260 K and 50–70 % relative humidity (Figure 3). The humidity was measured to be ≍1.0–1.3 mbar using a hygrometer sitting 20 cm above the cryo-sample holder. The temperature was increased in steps to 260 K within about four hours during which the sample was monitored using FT-IR spectroscopy.
